# Genome Sequence of Thermoactinomyces daqus H-18, a Novel Thermophilic Species Isolated from High-Temperature Daqu

**DOI:** 10.1128/genomeA.01394-14

**Published:** 2015-01-08

**Authors:** Su Yao, Youqiang Xu, Chunhui Xin, Ling Xu, Yang Liu, Hui Li, Jinxia Li, Jiwen Zhao, Chi Cheng

**Affiliations:** a China National Research Institute of Food and Fermentation Industries, Beijing, China; b Shandong Bandaojing Lt., Co., Shandong, China

## Abstract

Thermoactinomyces daqus H-18 is a new species of Thermoactinomyces isolated from high-temperature Daqu used in the fermentation of Bandongjing sesame-flavor liquor. Its genome was sequenced and assembled (3.44 Mb). The coding sequences (CDSs) that correlated to high-temperature tolerance were annotated. The metabolic pathways for the compounds responsible for flavor were also found.

## GENOME ANNOUNCEMENT

Daqu, a primary microbial source and traditional fermentation starter, has been used to produce certain foods, such as vinegar and Chinese liquor with an attractive flavor for thousands of years (1–3). The production of Bandongjing sesame-flavor liquor occurs through mixed-microbial fermentation, while the high-temperature Daqu provides the microorganisms in this process (4). The high-temperature Daqu plays an important role in the quality of liquor (4). In previous studies, we isolated a series of bacterial strains from the high-temperature Daqu of Bandongjing, Shandong province, China (our unpublished data), and one of the preponderant strains, designated Thermoactinomyces daqus H-18, is a novel species of the genus Thermoactinomyces (5). The analysis of the mechanism for high-temperature tolerance and the metabolic pathways of the flavor chemicals for strain H-18, combined with the studies of other strains isolated, would contribute to the understanding of the functions of high-temperature Daqu for the fermentation of Bandongjing sesame-flavor liquor. Therefore, the genome of strain H-18 was sequenced.

We announce here the draft genome of strain H-18. The genome sequence was determined by Illumina HiSeq 2000 (6), and a total of 7,513,692 reads for shotgun sequencing and 14,633,180 reads for paired-end sequencing were produced. These reads were assembled using the CLC Genomics Workbench 5.0.1 system (CLC bio, Aarhus, Denmark) into 86 contigs (>500 bp) with a length of 3,441,572 bp and a G+C content of 48.8%. The genome sequence was annotated by the RAST server (7). tRNAs and rRNAs were analyzed by tRNAscan-SE version 1.23 (8) and RNAmmer 1.2 (9), respectively.

The genome sequence of strain H-18 contains 4,024 predicted protein-coding sequences (CDSs). Fifty-eight tRNAs and two rRNAs were identified. The CDSs for DnaK (two), DnaJ (one), and GrpE (two) were annotated, which are chaperones capable of repairing heat-induced protein damage (10). Another six CDSs for heat shock proteins were annotated, which are also responsible for high-temperature tolerance (11). The complete metabolic pathways were annotated for the production of acetate, acetaldehyde, propanoate, and butanoate, which contribute to the flavor of Bandongjing liquor. We also annotated 53 CDSs for peptidase and seven CDSs for amino acid dehydrogenase, which can degrade proteins and peptides into amino acids and produce ammonium from amino acids, respectively. Ammonium was found to be the precursor of tetramethylpyrazine (12). Tetramethylpyrazine was reported to have nutty, roasty, and toasty tonalities (13). The genome sequence and annotation of T. daqus H-18 might be helpful for improving the understanding of its tolerance to high temperature as well as contributing to the production of Bandongjing sesame-flavor liquor during the fermentation processes.

### Nucleotide sequence accession numbers.

This whole-genome shotgun project has been deposited at DDBJ/EMBL/GenBank under the accession no. JPST00000000. The version described in this paper is version JPST01000000.

## References

[1] Xiu L, Kunliang G, Hongxun Z 2012 Determination of microbial diversity in Daqu, a fermentation starter culture of Maotai liquor, using nested PCR-denaturing gradient gel electrophoresis. World J Microbiol Biotechnol 28:2375–2381. doi:10.1007/s11274-012-1045-y. 22806111

[2] Yan Z, Zheng XW, Han BZ, Han JS, Nout MJ, Chen JY 2013 Monitoring the ecology of *Bacillus* during Daqu incubation, a fermentation starter, using culture-dependent and culture-independent methods. J Microbiol Biotechnol 23:614–622. doi:10.4014/jmb.1211.11065. 23648849

[3] Zheng XW, Yan Z, Han BZ, Zwietering MH, Samson RA, Boekhout T, Robert Nout MJ 2012 Complex microbiota of a Chinese “Fen” liquor fermentation starter (Fen-Daqu), revealed by culture-dependent and culture-independent methods. Food Microbiol 31:293–300. doi:10.1016/j.fm.2012.03.008. 22608236

[4] Wang HY, Gao YB, Fan QW, Xu Y 2011 Characterization and comparison of microbial community of different typical Chinese liquor Daqus by PCR-DGGE. Lett Appl Microbiol 53:134–140. doi:10.1111/j.1472-765X.2011.03076.x. 21554340

[5] Yao S, Liu Y, Zhang M, Zhang X, Li H, Zhao T, Xin C, Xu L, Zhang B, Cheng C 2013 *Thermoactinomyces daqus* sp. nov., a thermophilic bacterium isolated from high temperature Daqu. Int J Syst Evol Microbiol 64:206–210. doi:10.1099/ijs.0.055509-0. 24048866

[6] Liu L, Li Y, Li S, Hu N, He Y, Pong R, Lin D, Lu L, Law M 2012 Comparison of next-generation sequencing systems. J Biomed Biotechnol 2012:251364. doi:10.1155/2012/251364. 22829749 PMC3398667

[7] Aziz RK, Bartels D, Best AA, DeJongh M, Disz T, Edwards RA, Formsma K, Gerdes S, Glass EM, Kubal M, Meyer F, Olsen GJ, Olson R, Osterman AL, Overbeek RA, McNeil LK, Paarmann D, Paczian T, Parrello B, Pusch GD, Reich C, Stevens R, Vassieva O, Vonstein V, Wilke A, Zagnitko O 2008 The RAST server: Rapid Annotations using Subsystems Technology. BMC Genomics 9:75. doi:10.1186/1471-2164-9-75. 18261238 PMC2265698

[8] Lowe TM, Eddy SR 1997 tRNAscan-SE: a program for improved detection of transfer RNA genes in genomic sequence. Nucleic Acids Res 25:955–964. doi:10.1093/nar/25.5.0955. 9023104 PMC146525

[9] Lagesen K, Hallin P, Rødland EA, Staerfeldt HH, Rognes T, Ussery DW 2007 RNAmmer: consistent and rapid annotation of ribosomal RNA genes. Nucleic Acids Res 35:3100–3108. doi:10.1093/nar/gkm160. 17452365 PMC1888812

[10] Schröder H, Langer T, Hartl FU, Bukau B 1993 DnaK, DnaJ and GrpE form a cellular chaperone machinery capable of repairing heat-induced protein damage. EMBO J 12:4137–4144. 7900997 10.1002/j.1460-2075.1993.tb06097.x PMC413706

[11] Parsell DA, Lindquist S 1993 The function of heat-shock proteins in stress tolerance: degradation and reactivation of damaged proteins. Annu Rev Genet 27:437–496. doi:10.1146/annurev.ge.27.120193.002253. 8122909

[12] Zhu BF, Xu Y 2010 A feeding strategy for tetramethylpyrazine production by *Bacillus subtilis* based on the stimulating effect of ammonium phosphate. Bioprocess Biosyst Eng 33:953–959. doi:10.1007/s00449-010-0419-5. 20306320

[13] Masuda H, Mihara S 1988 Olfactive properties of alkylpyrazines and 3-substituted 2-alkylpyrazines. J Agric Food Chem 36:584–587. doi:10.1021/jf00081a044.

